# CARER program for autism spectrum disorder: a formative qualitative study on developing an early play-based, parent-mediated intervention in the Indian context

**DOI:** 10.1186/s13034-026-01027-2

**Published:** 2026-01-16

**Authors:** Arun Singh Yadav, Lakshmi Sravanti, Vinay Singh Chauhan, Harpreet Singh, Arul Velusamy, Rajendra Kiragasur Madegowda

**Affiliations:** 1 426 Field Hospital, Jammu, India; 2 Department of Child and Adolescent Psychiatry, National Institute of Mental Health and Neurosciences (NIMHANS), Bengaluru, India; 3 Military Hospital, Mhow, Indore, India; 4 Department of Psychiatry, Maharishi Markandeshwar University (MMU), Solan, India; 5 Oxfordshire Eating Disorders Pathway Team, Oxford, UK

**Keywords:** Autism, Autism spectrum disorder (ASD), NDBI, Parent-mediated intervention

## Abstract

**Background:**

Families of children with Autism Spectrum Disorder (ASD) often face unmet needs in psychoeducation, skill-building, and coping with behavioral challenges, particularly in low-resource or task-sharing settings. Existing parent-mediated interventions are either intensive, specialist-led, or focus primarily on psychoeducation, leaving gaps in structured caregiver training and support for parental well-being. Therefore, we aimed to develop an early, play-based, parent-mediated intervention program integrating Naturalistic Developmental Behavioral Interventions (NDBI) and structured play-based strategies to enhance caregiver competence and child developmental outcomes, tailored for use in resource-scarce, brief outpatient settings.

**Methods:**

The current study reports the qualitative phase of a broader mixed-methods, proof-of-concept investigation conducted at a premier medical college and its affiliated tertiary hospital within the Armed Forces Medical Services (AFMS), India. Focus group discussions were conducted with purposively selected primary stakeholders (five professionals and five parents of children ASD) supplemented by expert validation to develop a play-based, parent-mediated intervention. Only qualitative findings from the program development phase are presented; quantitative feasibility and outcome data will be reported separately. Thematic analysis of detailed field notes informed program adaptation. The CARER (Communication & social skills, Autism, Restricted and repetitive behaviors management, Empowerment of caregivers, and Responsive play) intervention program was structured into 12 outpatient sessions (45–60 min each), incorporating psychoeducation, modeling, guided parent–child practice, barrier-solving, home tasks, and strategies addressing parental stress. Credibility was ensured through investigator triangulation and member checking, with reporting aligned to Consolidated Criteria for Reporting Qualitative Research (COREQ) guidelines.

**Results:**

Thematic analysis to understand stakeholders’ perspectives revealed four core domains: (i) psychoeducation, (ii) caregiver training needs, (iii) educational needs of the child, and (iv) parental stress. The CARER program operationalizes these themes into structured, play-based sessions targeting communication, social interaction, restricted and repetitive behaviors, sensory issues, and caregiver empowerment. The program emphasizes parent-mediated delivery and home generalization of skills, balancing feasibility in outpatient settings with developmental relevance for children with ASD.

**Conclusions:**

The CARER program represents an early, brief, and pragmatically designed outpatient parent-mediated intervention framework to support families of young children with ASD. Further piloting and systematic evaluation in larger samples across similar low-resource settings are needed to assess feasibility, acceptability, fidelity of delivery, and potential clinical impact, and to inform ongoing adaptation.

*Trial registration*: AFMRC PROJECT NO :5337 /2020

**Supplementary Information:**

The online version contains supplementary material available at 10.1186/s13034-026-01027-2.

## Background

Children of Indian Armed Forces (IAF) personnel constitute 33% of the clientele of health services [1]. While the prevalence of ASD is rising [2], exact numbers within IAF is not known. Military families face high stress levels due to their lifestyle, and those raising a child with autism spectrum disorder (ASD) face additional challenges due to limited accessibility to services and difficulty in sustaining interventions [3]. Continuity of care is crucial for children with ASD, however, due to the transient nature of military postings, these families often lack access to multidisciplinary clinics that provide holistic early interventions, making service delivery even more challenging. Moreover, there is a significant gap between demand and supply in this field [4], with resource-poor settings often lacking the infrastructural and human resource support necessary to deliver structured play-based interventions effectively.

Research evaluating parent-mediated interventions in low-resource settings is on the rise and there is a need to adapt interventions to fit the needs of culturally diverse families [5]. Multiple studies in India and neighbouring Low-Middle Income Countries (LMICs) have explored various early intervention models for children with autism spectrum disorder (ASD), including center-based, home-based, and parent-mediated approaches. Programs such as Communication Developmental Eclectic Approach to Language Learning (ComDEALL) [6], Parent-mediated Autism Social communication intervention for non-Specialists **(**PASS) [7], and other brief parent-mediated interventions demonstrated improvements in child developmental domains, communication, and autism symptoms, along with reduced parental stress. These interventions varied in intensity, delivery agents (specialists vs. non-specialists), and settings, but consistently emphasized the importance of family involvement and feasibility in low-resource environments. These studies indicate that even parents with lower socio-economic status and educational levels are also able to implement the interventions effectively. Table 1 summarizes nature and key components of various studies done in India.

**Table 1:** Summary of the nature and key components of studies conducted in India

Study	Setting	Type of intervention	Target group (age & sample size)	Key components	Duration
Malhotra et al. [8]	Hospital-based	Psychological interventions for parents	Not specified	Supportive techniques vs. information	-
Karanth et al. [6]	Center-based	Intensive specialist-led	< 6 years; *n* = 30	Communication DEALL Checklist, 8 domains	8 months
Juneja et al. [9]	Hospital-based	Outpatient-based parent training	< 6 years	Inputs for home training	6 months
Nair et al. – Hospital-based [10]	Hospital	Low-intensity center-based	Mean age 36 months; *n* = 39	Parent training + clinical + school advice	-
Nair et al. – Home-based [11]	Home + clinic	Low-intensity home-based	Mean age 36 months; *n* = 52	Parent training + home practice + follow-up	~ 6 months
Patra et al. [12]	Outpatient hospital	Group psychoeducation for parents	Parents of children with ASD	Info about ASD, stress management	-
Rahman et al. [7]	Community, LMICs (India, Pakistan)	Parent-mediated by non-specialists	2–9 years; PASS + TAU (*n* = 32) vs. TAU (*n* = 33)	PASS intervention (based on PACT: Pre-school Autism Communication Therapy),	6 months (12 sessions)
Manohar et al. [13]	Hospital (low-resource)	Brief parent-mediated home-based	2–6 years; Intervention group (*n* = 26) vs. active control group (*n* = 24)	NDBI approach (focus on joint attention, imitation, social and adaptive skills)	12 weeks (5 sessions)

Abbreviations used in the table – ComDEALL: Communication Developmental Eclectic Approach to Language Learning; NDBI: Naturalistic Developmental Behavioral Interventions; PACT: Pre-school Autism Communication Therapy; PASS: Parent-mediated Autism Social communication intervention for non-Specialists; TAU: Treatment As Usual

A proof-of-principle pilot study evaluated the feasibility of a 12-session cascaded task-sharing Naturalistic Developmental Behavioural Intervention (NDBI) for young children with autism and their caregivers. Results demonstrated improvements in caregiver and non-specialist fidelity, as well as significant gains in child developmental and adaptive outcomes and reductions in caregiver stress. These findings support the potential of task-sharing NDBI models in low-resource settings, warranting further investigation through larger-scale studies [8]. The COMmunication-centred Prent-mediated treatment for Autism Spectrum Disorder in South Asia (COMPASS) trial is currently underway. This multi-site randomized controlled trial evaluates the clinical and cost-effectiveness of the ‘PASS Plus’ parent-mediated intervention, adapted for delivery by non-specialist health workers within the public health system in India. If proven effective, it holds promise as a scalable, contextually grounded model of care for low-resource settings [9].

Armed Forces Medical College (AFMC) and Command Hospital (CH) Pune are premier medical institutes in India having distinction for education and research. As part of Armed Forces Medical Services (AFMS), these institutes provide tertiary-level care to serving and veterans of Indian Armed Forces personnel and their dependents. Given the unique needs of this population, we undertook a broader mixed-methods proof-of-concept study to identify the unmet needs of families with a child diagnosed with autism with an aim to develop and pilot a parent-mediated intervention program integrating principles of Naturalistic Developmental Behavioral Interventions (NDBI) and play-based therapy for caregivers of children with autism. The primary objective of the qualitative phase of our study was to develop and formalize a structured, play-based, parent-mediated intervention program for children with Autism Spectrum Disorder (ASD). This paper focuses specifically on the process of intervention development and formalization, and does not address the quantitative phase, including pilot testing or quantitative outcomes.

## Methods

### Study design

A formative qualitative study employing a participatory research approach to intervention development. The study integrated methods such as Focus Group Discussions (FGDs) with parents and experts and expert validation to adapt and finalize a play-based parent-mediated intervention program.

### Study setting

The study was conducted at the out-patient services of Department of Psychiatry, AFMC and CH Pune (India) premier tertiary care centre catering to the families of Indian Armed Forces personnel. One Focus Group Discussion (FGD) with parents was conducted in-person at Armed Forces Medical College (AFMC) and Command Hospital (CH), Pune (India) while the professional FGD was conducted online via a secure video-conferencing platform.

### Participants and sampling strategy

 Purposive sampling was employed to recruit participants with direct experiential or professional expertise relevant to the development of a parent-mediated, play-based intervention for young children with Autism Spectrum Disorder within the Indian Armed Forces context. Sampling decisions were guided by the aim of capturing information-rich perspectives rather than achieving representativeness. Caregiver participants (*n* = 5) were recruited through outpatient services at the Department of Psychiatry, Armed Forces Medical College (AFMC) and Command Hospital (CH), Pune. Treating clinicians informed eligible caregivers about the study, after which interested participants were approached by the research team. Inclusion criteria included being a primary caregiver of a child diagnosed with ASD, active involvement in daily caregiving and intervention-related decision-making, and willingness to participate in a group discussion. Caregivers were selected to reflect families navigating service-linked contextual demands, including relocations and variable access to specialist services. Professional participants (*n* = 5) were identified through investigators’ professional networks and selected based on clinical and/or academic expertise in autism and child development, and experience within Indian public-sector or Armed Forces–linked healthcare systems. Invitations were extended via personal communication. Separate focus group discussions were conducted for caregivers and professionals to facilitate open discussion within peer groups and minimize power differentials, thereby supporting candid sharing of perspectives. In addition, we consulted one expert for final validation of the intervention program. Participants were approached through outpatient services of the Department of Psychiatry, AFMC and CH Pune (for parents) and via professional networks online (for clinicians/experts). Recruitment was done face-to-face and via personal invitations online. Participants were provided with detailed information sheets, along with consent and assent forms, which explained the purpose of the study, the researcher’s role, and the objectives of the research, ensuring transparency and informed participation. No participants dropped out of the study.

### Participant characteristics

 The caregiver focus group consisted of five primary caregivers of children diagnosed with ASD. All caregivers were parents and the primary decision-makers for their child’s care; this group included three mothers and two fathers to capture diverse caregiving perspectives. The children were in early childhood (2–6 years of age) and were receiving outpatient services at the study setting. Caregivers varied in employment status, including homemakers and those engaged in paid employment. All participating caregivers were proficient in Hindi, the language used for the focus group discussions, reflecting its widespread use across Armed Forces families due to the nature of postings and service-related mobility. The professional focus group comprised five experts from disciplines related to child development and autism, including child and adolescent psychiatry, developmental pediatrics, psychology, and special education. All professionals held formal qualifications in their respective fields and had substantial clinical or educational experience working with children with autism and their families, with individual experience ranging from 10 to 15 years (combined experience: 62 years). Most participants were engaged in public-sector or tertiary-care service delivery. Given the small sample size and the identifiable nature of the clinical setting, detailed individual-level demographic data are not reported to protect participant confidentiality, in keeping with ethical guidelines for qualitative research.

### Data collection

Focus Group Discussions (FGDs) were the primary method of data collection. Separate FGDs were conducted with (a) experts (e.g., psychiatrists, child psychiatrists, developmental pediatricians, special educators) and (b) parents of children diagnosed with autism. FGD with experts lasted 60 min and the FGD with caregivers was carried out for 90 min and was facilitated by the first author. No repeat sessions were held; each focus group participated once. Only the participants and the facilitator (the first author) were present, and no non-participant observers attended the discussions. FGDs were conducted using a semi-structured guide developed by the authors and reviewed for content relevance, though not formally pilot-tested. Sessions were not audio-recorded. Instead, detailed contemporaneous field notes were taken by the first author during each discussion and expanded immediately afterward to capture key discussion points, representative participant statements, group dynamics, and contextual observations. Caregiver focus group discussions were conducted in Hindi to facilitate comfort and nuanced expression. Field notes from these sessions were translated into English by the bilingual primary researcher and subsequently reviewed by another bilingual member of the research team to ensure conceptual accuracy and preservation of meaning. Non-verbal cues and emotional expressions documented in the field notes were incorporated into the analytic process. The facilitator adopted an open-ended, reflective interviewing style and engaged in ongoing reflexive practice to minimize interpretive bias during data collection and analysis. These data informed the identification of psychoeducational needs, caregiver training gaps, perceptions of play-based intervention, and contextual barriers to care. Reporting of the qualitative methods and findings adhered to the Consolidated Criteria for Reporting Qualitative Research (COREQ) guidelines [10].

###  Research team and reflexivity

#### Interviewer background

FGDs were facilitated by the first author (ASY), the principal investigator of the study, a qualified male child and adolescent psychiatrist with a master’s degree in Psychiatry (MD) and post-doctoral specialization (DM) in Child and Adolescent Psychiatry with experience in conducting qualitative research, and trained in moderating focus group discussions.

#### Role of the researcher

 The researcher’s role was to guide discussions, ensure all participants’ views were represented, and maintain a neutral stance without leading responses.

#### Researcher–participant relationship

 No prior therapeutic relationship existed between the researcher and caregiver participants. Professional participants were colleagues within the broader child development and autism field but did not have a supervisory or evaluative relationship with the researcher. Participants were informed of the facilitator’s role as a clinician-researcher and the study’s aim of developing a caregiver training program.

#### Participant knowledge of interviewer’s role or aims

 Participants were informed that the facilitator was a researcher developing a parenting intervention program for autism. The study objectives, facilitator’s role, and expectations from participants were explained prior to the FGDs.

#### Data saturation

Given the proof-of-concept nature, data saturation was not formally assessed. However, thematic convergence was observed within and across FGDs, and no new themes emerged in the latter half of discussions.

#### Data analysis

Thematic analysis was conducted on detailed field notes and verbatim excerpts documented during the focus group discussions with experts and parents. An inductive coding approach was used. Two independent researchers manually coded the data line by line and resolved differences through discussion and consensus. No qualitative analysis software was used. Initial open codes were generated to capture meaningful units of data reflecting participant perspectives. Similar codes were subsequently grouped into subcategories, which were then clustered into broader categories. Through an iterative process of refinement and comparison across data sources, these categories were consolidated into final minor themes and overarching major themes. A coding tree was developed to document the analytic progression from raw data excerpts to codes, subthemes, and final themes, providing a transparent and traceable representation of how specific participant statements informed each thematic outcome.

Beyond theme generation, the analytic process explicitly focused on translating qualitative findings into concrete program components. Each major theme and corresponding subthemes were systematically mapped onto key aspects of program development, including content areas (e.g., psychoeducation, play-based strategies), delivery formats (e.g., session structure, modeling, guided practice), and contextual adaptations (e.g., home-based feasibility, caregiver burden). Themes derived from caregiver FGDs primarily informed the identification of caregiver needs, perceived barriers to care, preferred modes of learning, and culturally relevant examples. In contrast, themes emerging from professional FGDs guided clinical priorities, sequencing of intervention components, and alignment with evidence-based practices. This mapping process was iterative and reviewed by the research team to ensure that each core program element could be traced back to one or more qualitative themes. The final intervention structure thus reflects a synthesis of experiential knowledge from caregivers, professional clinical expertise, and investigator-led adaptation of an existing evidence-based parent-mediated intervention model. Credibility of the analysis was strengthened through triangulation of data sources (i.e., caregivers, professionals, and peers) and member checking during the validation phase. To enhance transparency of the analytic process, a detailed coding tree illustrating the progression from data excerpts to final themes is provided as Supplementary File S1.

### Program development process

The following steps were undertaken to design the intervention program:

1. Model playroom (MP) setup: A dedicated Model Playroom was established within the Armed Forces Medical College premises, equipped with standard items and accessories suitable for children with special needs, providing a child-friendly and engaging environment for parent-child interaction.
2. Integration of FGD findings: Data from FGDs with experts and parents were systematically reviewed. Emerging themes were mapped onto essential content areas and structural elements for the intervention.
3. Adaptation of existing program: The National Institute of Mental Health and Neuro Sciences (NIMHANS) brief in-patient parent-mediated intervention model [11], was adapted for the context of Indian Armed Forces families. The adaptations emphasized play-based, home-friendly activities using readily available toys, ensuring cultural and contextual relevance.
4. Investigator expertise: The primary investigator and first author, trained at NIMHANS as a child psychiatrist with extensive hands-on experience in the NIMHANS brief in-patient parent-mediated intervention model, led the adaptation process. Having been primarily trained and working with Armed Forces families for over two decades, he identified a *felt need* for such a program in this context and ensured fidelity to the core principles of the original intervention while tailoring it to the unique requirements of Armed Forces families.
5. Expert validation: The draft program was reviewed by an independent expert for content validity, feasibility, and alignment with best practices. Feedback was incorporated to finalize the program.

### Ethical considerations

 Ethical approval was obtained from the Institutional Ethics Committee of the Armed Forces Medical College, India (AFMRC PROJECT NO :5337/2020). All procedures followed ethical standards for research involving human participants, with confidentiality and voluntary participation ensured throughout the study. Written informed consent was obtained from all participants.

## Results

Thematic analysis of FGDs with experts and parents yielded four overarching themes:

### Theme 1: psychoeducation (beyond diagnosis to meaning-making)

Caregivers’ narratives indicated that psychoeducation needs extended beyond understanding diagnostic labels to making sense of what autism meant for their child’s daily functioning, future development, and family life. Parents frequently described receiving a diagnosis without accompanying guidance on “what to do next,” resulting in uncertainty and emotional distress. Experts similarly noted that fragmented or inconsistent information often delayed help-seeking and early intervention. In the Armed Forces context, where families may relocate frequently and encounter variable service availability, the absence of clear, structured psychoeducation further compounded caregiver confusion. These findings informed the inclusion of a dedicated psychoeducation component within the CARER program that emphasized anticipatory guidance, practical goal-setting, and realistic expectations rather than purely diagnostic information.

### Theme 2: caregiver training needs (Move from “What” to “How”)

While training needs related to communication, social participation, and behavior management were anticipated, the analysis revealed that caregivers were not merely seeking information about *what* strategies to use, but guidance on *how* to apply these strategies within everyday routines. Parents described difficulty translating professional advice into home-based interactions, particularly during unstructured play. Experts similarly noted that caregiver confidence and consistency were often more limiting to progress than child-related factors. Both experts and parents highlighted gaps in practical skills for supporting communication, social participation, and regulation challenges in everyday contexts, particularly during early developmental periods when timely family-centered supports can enhance functional outcomes. This underscored the importance of a play-based, modeled, and guided practice approach in the CARER program, where parents actively rehearsed strategies rather than receiving didactic instruction.

### Theme 3: educational needs of the child (navigating readiness, access, and inclusion)

Parents articulated significant uncertainty and concern regarding their child’s educational pathway, particularly in relation to school readiness, access to appropriate educational supports, and inclusion within mainstream settings. Many caregivers described difficulty determining when and how to initiate schooling, given their child’s communication and regulation challenges, and expressed anxiety about whether schools would be equipped to meet their child’s needs. Parents frequently reported limited guidance on navigating special education services, identifying suitable schools, or advocating for classroom accommodations. Experts echoed these concerns, noting gaps in coordination between health and educational systems and limited autism-specific preparedness among teachers, especially in non-specialist or resource-constrained settings. Accordingly, the CARER program incorporated elements focused on supporting parents in understanding educational readiness, and aligning play-based developmental goals with early learning expectations.

### Theme 4: parental stress (contextual deepening)

Parental stress emerged not only from day-to-day caregiving demands but also from systemic and contextual factors inherent to service-linked family life. Regular relocations, which are an essential and expected aspect of Armed Forces service, were noted to influence continuity of developmental services, often necessitating changes in providers and settings. Parents described how this required repeated adjustment and re-orientation to services, contributing to fatigue, reduced confidence in caregiving strategies, and a sense of managing care with limited local supports at times. Recognizing these stressors shaped the program’s emphasis on portable, home-based strategies that could be sustained across settings, while reinforcing caregiver confidence and continuity of practice despite changes in service environments and reducing reliance on location-specific services.

The major themes and their corresponding minor themes are presented in Table 2, along with illustrative quotes.

**Table 2:** Major and minor themes with illustrative quotes

Major theme	Minor theme	Illustrative quote
1. Psychoeducation (beyond diagnosis to meaning-making)	Need for clarity on condition and prognosis	*“When my child was diagnosed*,* I only got a label. No one told me what this actually means for his future.”* (Parent)
	Awareness of early indicators	*“Families are often unaware of the early signs of autism; improving general awareness could enhance help-seeking and access to available interventions within the community.”* (Expert)
2. Caregiver training needs (Move from “What” to “How”)	Communication support needs	*“I don’t know how to make him talk. Sometimes I feel lost when he only points or cries.”* (Parent)
	Supporting social participation	*“He doesn’t know how to play with other children*,* and I don’t know how to guide him.”* (Parent)
	Behavior management strategies	*“Parents often ask not just about handling meltdowns but also about managing repetitive behaviors like lining up objects or insistence on routines in daily life.”* (Expert)
	Need for coaching over instruction	*“Everyone tells us what he should learn*,* but no one shows us how to actually do it with him at home.”* (Parent)
3. Educational needs of the child (navigating readiness, access, and inclusion)	Academic readiness concerns	*“Before school starts*,* we don’t even know if he is ready to sit in a classroom.”* (Parent)
	Need for special education support	*“Schools are not equipped. Teachers don’t understand what autism means in the classroom.”* (Parent)
	Integration with mainstream schooling	*“Parents want their children to be included*,* not sidelined*,* but the options are very few.”* (Expert)
4. Parental stress (contextual deepening)	Emotional burden	*“I keep wondering if I did something wrong for my child to have autism.”* (Parent)
	Lack of social support	*“Family and friends withdraw*,* and you are left to handle everything alone.”* (Parent)
	Balancing caregiving with other responsibilities	*“It is a daily struggle to manage therapy appointments*,* work*,* and home responsibilities.”* (Parent)
	Disrupted service continuity	*“Armed Forces families face frequent transfers*,* disrupting continuity of care.”* (Expert)

### CARER program: framework and implementation

Based on thematic findings and iterative adaptation, the final parent-mediated intervention was designated CARER (Communication & social skills, Autism, Restricted and repetitive behaviors management, Empowerment of caregivers, and Responsive play). The acronym reflects the dual focus of the program: addressing core developmental domains in children with autism including communication, social interaction, restricted and repetitive behaviors, and sensory challenges, while keeping the caregiver central to intervention delivery and explicitly supporting their well-being and skill development – hence the name CARER. Intervention components were refined iteratively through team discussions following thematic analysis, ensuring fidelity to participant narratives while maintaining feasibility for delivery in low-resource and primary-care settings. While the CARER domains align with the major themes identified in the qualitative analysis, they represent a pragmatic consolidation of caregiver needs and expert insights into deliverable intervention targets, rather than a direct thematic reproduction or one-to-one mapping.

This program integrates key strategies from Naturalistic Developmental Behavioral Interventions (NDBI) with play-based methods to enhance caregiver–child interaction. Training was delivered using a structured but flexible approach, incorporating psychoeducation and skill-building components. Psychoeducation introduced each theme, followed by modeling of play routines (e.g., turn-taking, joint attention, imitation, communication) and guided caregiver practice with feedback. Strategies were embedded into everyday routines, with home assignments to extend use beyond the clinic. Orientation to play-based intervention formed the foundation, followed by targeted strategies for:

- *Socialization: *Guided parent–child play routines addressing joint attention and peer readiness were developed in response to caregivers’ reported difficulties in supporting social participation and preparing children for group and classroom settings.
- *Communication:* Communication strategies emphasized modeling and guided practice, reflecting parents’ expressed need for coaching rather than purely instructional advice.
- *Connection interventions:* Connection-focused activities were included to strengthen affective attunement and shared enjoyment, addressing caregiver stress, emotional uncertainty, and the need for meaningful parent–child interaction identified across themes.
- *Restricted and repetitive behaviors (RRB) and sensory issues management:* Strategies for managing restricted and repetitive behaviors and sensory sensitivities were derived from caregiver concerns about daily routines and behavior regulation, with an emphasis on practical, context-sensitive adaptations such as – identification of triggers, environmental modifications, and redirection or reinforcement strategies tailored to each child’s needs.

### Illustrative examples of intervention components

To enhance clarity regarding the nature of the intervention, selected examples of strategies used within each component are described below. These examples are illustrative rather than exhaustive and are adapted to be culturally and contextually feasible within Indian family settings.

- *Socialization: *Guided parent–child play routines were used to build foundational social skills such as eye contact, joint attention, imitation, and turn-taking. For example, caregivers were coached to engage the child in simple turn-taking games (e.g., rolling a ball back and forth or building a block tower together) while sitting at the child’s eye level and following the child’s lead. Visual activity schedules were also introduced to prepare children for transitions during daily routines (e.g., getting ready to leave for school), thereby reducing anxiety and promoting social readiness.
- *Communication:* Communication strategies focused on creating natural opportunities for functional communication within play and daily routines. For instance, caregivers were encouraged to keep preferred toys just out of reach during play to prompt requesting through eye contact, pointing, vocalization, or words. Parallel talk (describing the child’s actions during play) and modeling meaningful language (e.g., expanding a child’s vocalization into a functional phrase) were routinely practiced during activities such as pretend cooking, doll play, or book reading.
- *Connection interventions:* Connection-focused activities aimed to enhance affective attunement, shared enjoyment, and caregiver–child bonding through structured, playful interactions. Examples included therapist-guided dyadic games such as turn-based movement games, sensory play (e.g., sand or bubble play), and reciprocal body-based games that emphasized positive touch, shared laughter, and mutual engagement. Caregivers were supported to replicate these activities at home to strengthen emotional connection in naturalistic settings.
- *Restricted and repetitive behaviors (RRB) and sensory issues management:* Interventions targeted both understanding and managing RRBs and sensory sensitivities. For example, caregivers were trained to identify triggers for stereotyped behaviors and to redirect these behaviors into shared, meaningful play (e.g., transforming repetitive lining up of toys into a pretend play scenario). For inflexibility related to routines, visual schedules were introduced and gradually modified to promote tolerance of change. Sensory strategies included creating a simple sensory play space at home and using practical accommodations (e.g., headphones for auditory sensitivity) alongside gradual exposure to sensory experiences.

Together, these examples illustrate how the CARER Program translates core principles of Naturalistic Developmental Behavioral Interventions into concrete, caregiver-delivered strategies embedded within everyday routines. A detailed description of the CARER intervention, including its session domain–level structure and corresponding caregiver and therapist objectives, is provided in Supplementary File S2.

### Session structure and duration

 Each 45–60 min session included psychoeducation, live demonstration, caregiver practice with feedback, and discussion of challenges. Home practice tasks reinforced learning, and brief check-ins on parental stress and coping supported caregiver well-being.

### Number of sessions

The program comprises 12 sessions, with flexibility to adjust based on the child’s baseline functioning and caregiver readiness. Additional booster sessions may be incorporated as needed. The 12-session structure was chosen in line with parent-mediated NDBI models (10–16 sessions), balancing adequate exposure and practice with caregiver feasibility in low-resource outpatient settings.

### Individualization of training

The program allows individualization of content and intensity based on the child’s baseline level of functioning, assessed by standard rating scales/checklists. This ensures that priority areas such as social, communication, behavioral regulation, or sensory domains are addressed according to the child’s profile and the caregiver’s expressed concerns. However, the core CARER components – including psychoeducation, guided modeling of caregiver–child interactions, and structured home-practice assignments represent non-negotiable elements to maintain intervention fidelity. Within these parameters, providers may tailor activities to individual child needs, available toys, and family routines. This balance between fidelity and adaptability allows the program to accommodate cultural, resource, and context-specific variations while preserving the essential principles of a play-based, parent-mediated intervention.

### CARER manual for clinicians

**Fig. 1:**
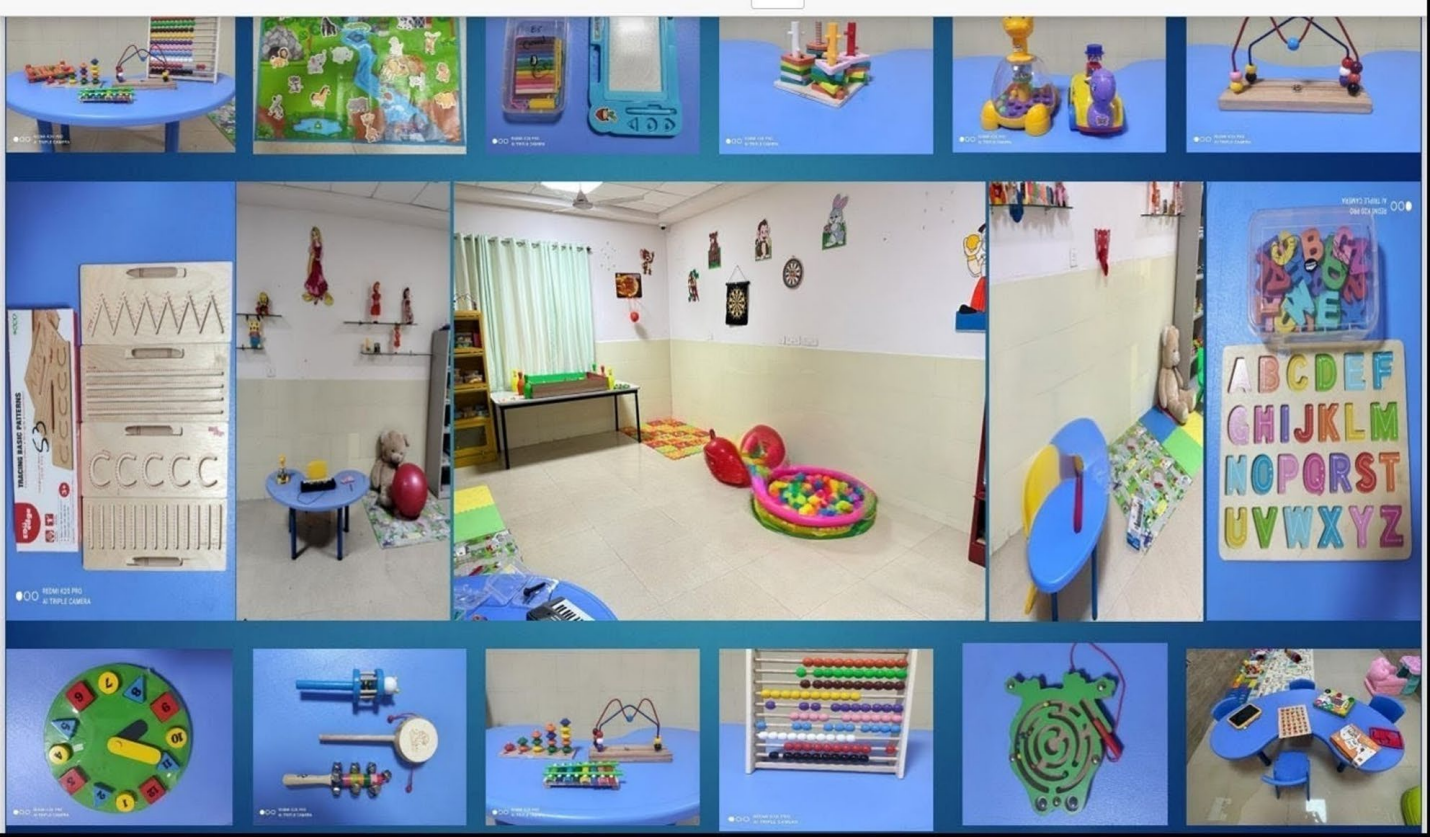
Collage of the prototype model playroom setup, illustrating the overall layout and the range of developmentally appropriate toys used to support social, communication, and sensory interventions in the CARER program

**Fig. 2:**
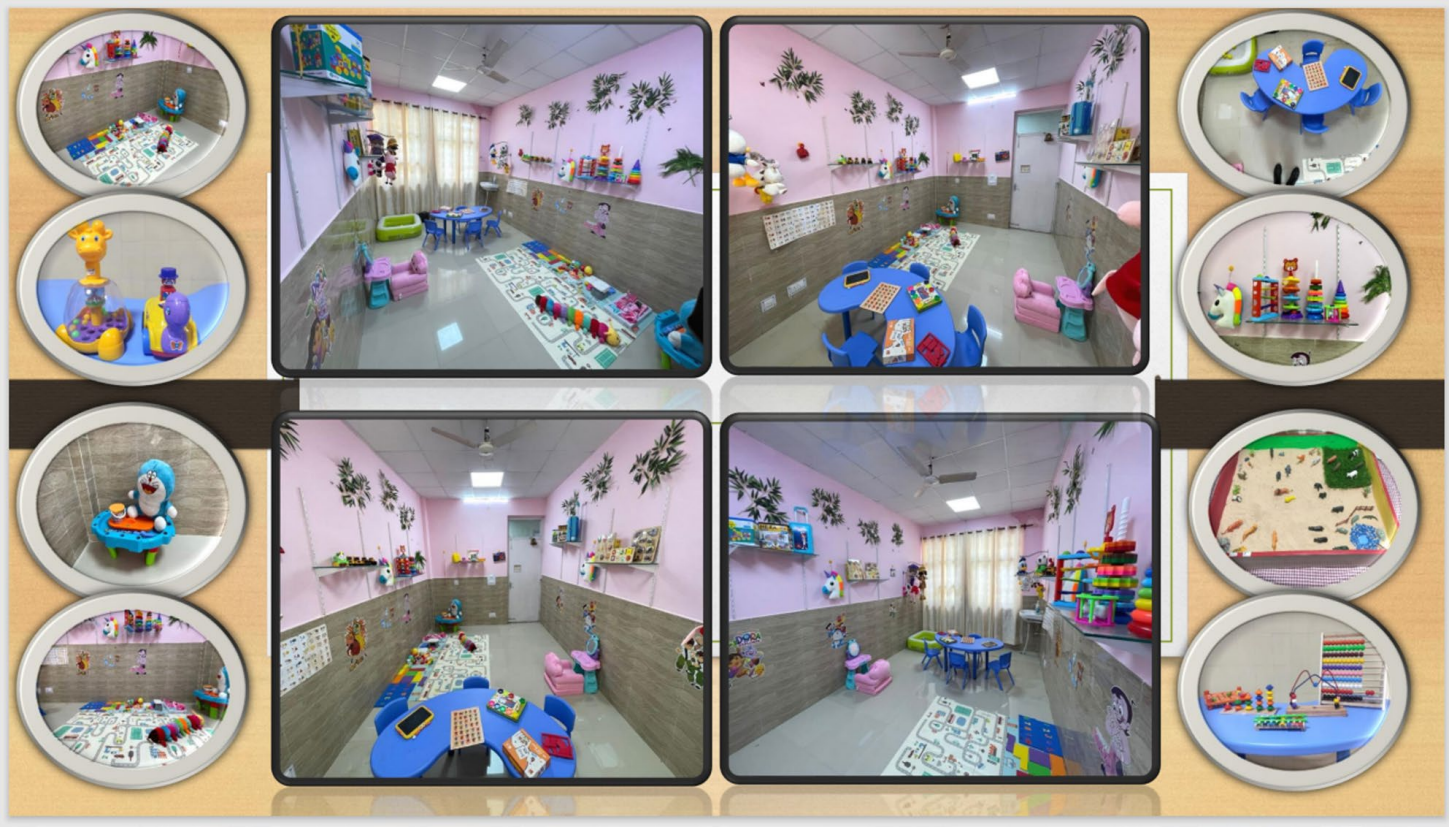
Collage of an alternative model playroom setup in a resource-limited clinical setting, highlighting the spatial arrangement and variety of toys designed for structured play-based interventions targeting social, communication, and restricted/repetitive behaviors in the CARER program

The clinician manual accompanying the CARER program provides a structured framework for implementation, aimed at standardizing delivery and enhancing clinician competence in promoting early awareness and intervention. It begins with the theoretical underpinnings and guiding principles, followed by practical guidance on selecting developmentally appropriate play materials and establishing a play therapy room suited to resource-limited settings. Subsequent chapters outline caregiver psychoeducation, orientation to play-based intervention, assessment procedures, and session structure and schedule. Stepwise modules detail interventions for socialization, communication, emotional connection, and management of restricted and repetitive behaviors and sensory issues. Supplementary sections include images of a prototype play-based intervention lab, standardized toy lists, and a tracking sheet to monitor caregiver implementation and child progress. Figures 1 and 2 illustrate model playroom setups and the range of developmentally appropriate toys included in the CARER program. To support replication and implementation, a detailed inventory of intervention materials, including therapeutic purpose, approximate cost ranges, and session-level applications, is provided in Supplementary File S3.

## Discussion

The CARER program was deliberately designed to be simple and caregiver-friendly, allowing delivery by non-specialists in low-resource and task-sharing contexts. This approach was informed by the substantial unmet needs of families in settings where infrastructural limitations, scarcity of trained professionals, and lack of standardized guidelines restricting access to individualized interventions. Given the unique practical constraints of armed forces families, the program was developed for feasible delivery in outpatient settings, with a focus on developmentally appropriate play-based activities and parent-mediated strategies to ensure skill generalization at home. Evidence from related contexts supports the utility of such an approach. An annual audit of families of children with ASD admitted to the in-patient facility at NIMHANS, Bengaluru, indicated that nearly 50% of parents reported some improvement in their child’s functioning at the time of discharge, suggesting the effectiveness of brief, structured interventions during admission [11]. Similarly, Vijayarani et al. [18], demonstrated that provision of an educational booklet to families of children with ASD significantly improved caregiver knowledge regarding management and promoted a more positive orientation towards childrearing [12]. These findings collectively substantiate the rationale for incorporating focused, parent-mediated, low-intensity programs that can be realistically implemented in resource-constrained settings while addressing the contextual needs of families.

The CARER program addresses contextual needs reported by families—psychoeducation and early-intervention awareness, practical caregiver skill-building for communication and behavior, school navigation, and attention to parental stress. These needs were shaped by families’ lived experiences of navigating care across varied service settings, differences in availability of specialist resources, and the need for continuity in caregiving practices despite changes in location or providers – realities that are characteristic of, but not unique to, Armed Forces families. Rather than framing these contextual realities as limitations, the CARER program was intentionally designed to work within such systems. These findings informed the development of a highly simplified yet scientifically grounded, play-based, parent-mediated program that emphasizes portability, consistency of core principles, and ease of delivery across settings. The program was structured so that it can be implemented not only by child development specialists but also by general medical practitioners (MBBS) and pediatricians, who are often the first – and sometimes the only point of care in remote or resource-limited contexts. As such, while informed by the Armed Forces context, the CARER program is designed for broader applicability in community and primary-care settings where specialist services may be limited or intermittently available. While remaining brief and outpatient-feasible, this profile bridges the gap between intensive, specialist-led centre programs [6], and information-focused or group psychoeducation models [13, 14], by combining hands-on coaching with structured content relevant to everyday care and educational decisions.

The present program, designed for low-resource outpatient settings is similar to previous low-intensity hospital- or home-based parent-training programs [15–18]. However, it extends prior work by combining NDBI and play-based strategies within a structured 45–60 min session format (psychoeducation → modeling → guided practice → barrier-solving → home tasks), incorporating explicit components addressing parental stress and coping, and emphasizing parent-mediated skill generalization. Its 12-session arc aligns with the effective range reported for parent-mediated models [7], balancing task-sharing feasibility with skills-focused delivery, while remaining shorter than intensive specialist-led programs [6], and more comprehensive than psychoeducation-only interventions [13, 14].

### Strengths, limitations and future directions

A major strength of the present study lies in its focus on developing a simple, structured, and caregiver-friendly intervention program intended for use in low-resource contexts. By drawing on principles of naturalistic developmental behavioral interventions (NDBI) and play-based approaches, the program translates evidence-informed strategies into an accessible format that can be integrated into routine outpatient care. The explicit inclusion of parental stress and coping reflects a holistic, family-centered orientation that is often underrepresented in intervention development.

Several limitations merit consideration. The program was developed within a single clinical setting, which may not fully capture the sociocultural, linguistic, and service-level diversity across India. Although the caregiver focus group included both mothers and fathers, fathers’ perspectives may be underrepresented because they spoke less during discussions, potentially limiting insights into paternal caregiving experiences. While the intervention was designed to be deliverable by non-specialist or primary-care providers, this has not yet been empirically tested, and fidelity of delivery in routine services may be influenced by variations in training, supervision, and infrastructure. The deliberately brief session structure, while suited to outpatient settings, may limit depth of skill acquisition for families requiring more intensive or sustained support. Finally, the present work does not include acceptability data, or child developmental outcomes, all of which require systematic evaluation in future phases of research.

The next planned step was to systematically evaluate the CARER program as an early intervention, given that the play-based approach is most suitable for young children and that thematic analysis highlighted caregivers’ and professionals’ expressed need for early-targeted supports. Proof-of-concept quantitative piloting has been undertaken; however, those findings are beyond the scope of the present paper and will be reported separately. Future research should extend this work by examining feasibility, acceptability, and preliminary outcomes across diverse service settings, including primary care and community-based programs, to assess adaptability and scalability. Comparative studies with existing parent-mediated interventions in the Indian context could further delineate the relative advantages of this approach.

## Conclusions

A structured, caregiver-friendly program informed by NDBI and play-based principles was developed to address the identified needs of families of young children with autism in low-resource outpatient settings. By adapting established intervention principles into a brief, outpatient-suited format, the CARER program represents a pragmatically designed, context-sensitive model of parent-mediated support. Further empirical work is required to evaluate feasibility, acceptability, fidelity of delivery, and clinical impact across diverse service settings and populations.

## Supplementary Information

Supplementary Material 1.

Supplementary Material 2.

Supplementary Material 3.

Supplementary Material 4.

## Data Availability

The datasets (interview transcripts) generated and analyzed during the current study are not publicly available, as permission for public sharing of raw data was not included in the participant consent form.

## Abbreviations

AFMC: Armed Forces Medical College
AFMRC: Armed Forces Medical Research Committee
AFMS: Armed Forces Medical Services
ASD: Autism Spectrum Disorder
CARER: Communication & social skills, Autism, RRB management, Empowerment of caregivers, and Responsive play
CH: Command Hospital
ComDEALL: Communication Developmental Eclectic Approach to Language Learning
COMPASS: Communication-centred Parent-mediated treatment for Autism Spectrum Disorder in South Asia
COREQ: Consolidated Criteria for Reporting Qualitative Research
DM: Doctorate of Medicine
FGD: Focus Group Discussion
FGDs: Focus Group Discussions
IAF: Indian Armed Forces
LMICs: Low-Middle Income Countries
MD: Doctor of Medicine
NDBI: Naturalistic Developmental Behavioural Intervention
NIMHANS: National Institute of Mental Health and Neuro Sciences
PACT: Pre-school Autism Communication Therapy
PASS: Parent-mediated Autism Social communication intervention for non-Specialists
RRB: Restricted and Repetitive Behaviors
TAU: Treatment As Usual
