# Supplementary material for: CARER program for autism spectrum disorder: a formative qualitative study on developing an early play-based, parent-mediated intervention in the Indian context
Source: Child Adolesc Psychiatry Ment Health. 2026 Jan 16;20:19. doi: 10.1186/s13034-026-01027-2 (PMC12892815; doi:10.1186/s13034-026-01027-2)
Supplement: Supplementary file 2 — Supplementary Material 2. [file 13034_2026_1027_MOESM2_ESM.docx]

**CARER Program for Autism Spectrum Disorder: A Formative Qualitative Study on Developing an Early Play-Based, Parent-Mediated Intervention in the Indian Context.**

**CODING TREEE ILLUSTRATING PROGRESSION FROM DATA EXCERPTS TO FINAL THEMES**

| **Example Data Excerpt (FGDs)** | **Initial Code** | **Minor Theme** | **Major Theme** |
| --- | --- | --- | --- |
| *“When my child was diagnosed, I was only told the label. No one explained what it really means or what to expect.” (Parent)* | Lack of diagnostic explanation | Need for clarity on condition and prognosis | Psychoeducation |
| *“Families often come very late because they don’t recognize early signs or don’t know where to seek help. Therefore, improving general awareness could enhance help-seeking and access to available interventions within the community.” (Expert)* | Limited awareness of early signs | Awareness of early indicators | Psychoeducation |
| *“I don’t know how to make him talk. Sometimes he just cries and I feel helpless. He points to things and I am unable to comprehend what he is trying to say. I feel completely lost.” (Parent)* | Caregiver uncertainty about communication | Communication support needs | Training Needs |
| *“Parents frequently ask how to manage everyday behaviors, not just meltdowns but repetitive routines too like lining up objects or insistence on routines in daily life.” (Expert)* | Difficulty managing behaviors | Behavior management strategies | Training Needs |
| *“He doesn’t play with other children. I keep trying but I fail. I don’t know how to teach him to interact.” (Parent)* | Challenges in peer interaction | Supporting social participation | Training Needs |
| *“After the diagnosis, many people explained what autism is and what my child is not doing yet. I read things online also. But when I sit with him at home, I don’t know what exactly I should do. They tell me ‘encourage communication’ or ‘play with him’, but no one shows me how to play or how to respond when he doesn’t talk. I feel stuck because I know the information, but I don’t know how to use it in real life.” (Parent)* | Difficulty translating information into action, therefore parents wanting “how-to,” not information | Need for coaching over instruction | Training Needs |
| *“Before school starts, we don’t even know if he can sit and learn like other children. We are clueless if we can enrol him in playschool or not.” (Parent)* | Concerns about school readiness | Academic readiness concerns | Educational Needs of the Child |
| *“We are constantly trying to find the right setup for my kid to learn. But teachers don’t seem to be trained to handle autism. We are not able to find appropriate schools.” (Parent)* | Lack of inclusive education support | Need for special education support | Educational Needs of the Child |
| *“Parents want inclusion, but mainstream schools often lack resources. The available options have limited support system.” (Expert)* | Barriers to inclusion | Integration with mainstream schooling | Educational Needs of the Child |
| *“I keep blaming myself, wondering if I did something wrong for my child to have autism I feel guilty all the time.” (Parent)* | Parental guilt | Emotional burden | Parental Stress |
| *“After diagnosis, friends and relatives slowly stop visiting. You are left to handle everything on your own.” (Parent)* | Social isolation | Lack of social support | Parental Stress |
| *“It is exhausting to manage therapy, work, and day-to-day home chores. The therapy required is intensive and time-consuming.” (Parent)* | Caregiver overload | Balancing caregiving with other responsibilities | Parental Stress |
| *“Armed Forces families face frequent transfers, disrupting continuity of care. The needs of families vary as their child grows and the availability of services is not uniform across the country. All these factors play a role in accentuating the already existing demand-supply gaps.” (Expert)* | Disrupted service continuity | Contextual caregiving stressors | Parental Stress |

**Table S1: Coding Tree illustrating progression from data excerpts to final themes**

**Note on Analytic Process**

This coding tree illustrates the inductive analytic pathway used in the study. Raw data excerpts from focus group discussions were first assigned initial codes, which were then grouped into minor themes. These minor themes were further consolidated into four overarching major themes. The coding tree was used as an analytic audit trail to ensure transparency and traceability between participant accounts and final thematic outcomes.
