# Supplementary material for: CARER program for autism spectrum disorder: a formative qualitative study on developing an early play-based, parent-mediated intervention in the Indian context
Source: Child Adolesc Psychiatry Ment Health. 2026 Jan 16;20:19. doi: 10.1186/s13034-026-01027-2 (PMC12892815; doi:10.1186/s13034-026-01027-2)
Supplement: Supplementary file 3 — Supplementary Material 3. [file 13034_2026_1027_MOESM3_ESM.docx]

**CARER Program for Autism Spectrum Disorder: A Formative Qualitative Study on Developing an Early Play-Based, Parent-Mediated Intervention in the Indian Context.**

**CARER INTERVENTION: SESSION DOMAIN-LEVEL STRUCTURE**

| **CARER Domain(s)** | **Therapist Objective** | **Caregiver Objective** | **Child-Focused Target** | **Child-Focused Target** | **Home-Practice Task** |
| --- | --- | --- | --- | --- | --- |
| Psychoeducation | Orient caregivers to program goals, establish rapport, clarify expectations | Understand autism and the CARER program | Familiarity with routines | Orientation using visual aids and discussion | Identify daily routines suitable for play-based interaction |
| Socialization | Guide caregivers in scaffolding social play, monitor child responses | Foster joint attention and peer readiness | Respond to social cues | Respond to social cues | Structured play sessions with sibling/peer |
| Communication | Model and coach turn-taking, prompting, and reinforcement strategies | Learn strategies to elicit functional language | Initiate requests, gestures, or words | Modeling of turn-taking and prompting strategies | Practice daily request-making during meals or play |
| Connection Interventions | Demonstrate attunement, shared enjoyment, and imitation strategies | Strengthen parent-child bonding and affective attunement | Shared enjoyment, emotional reciprocity | Activities promoting imitation and shared attention | Daily 5–10 min “connection play” using favorite toys |
| RRB & Sensory Management | Observe child behavior, coach redirection, environmental modifications | Identify and manage repetitive behaviors | Reduce distress from transitions or sensory overload | Environmental modifications, redirection strategies | Track triggers and implement home-based coping strategies |
| Integrated Practice | Support caregiver problem-solving, troubleshoot challenges | Combine all learned domains | Functional application in routines | Structured play incorporating communication, social, and RRB strategies | Apply multi-domain strategies during bath, meal, or outdoor play |
| Consolidation / Booster Sessions | Monitor caregiver fidelity, provide corrective feedback, ensure consistency | Reinforce caregiver skills, troubleshoot challenges | Consistency of application | Review and guided practice of prior strategies | Continue daily practice with adjustments |

**Table S2: CARER Intervention: Session Domain-level Structure**

**Notes:**

- Format: Individual sessions
- Number of sessions: 12 (flexible to child baseline and caregiver readiness), with optional booster sessions
- Each session lasts 45–60 minutes and includes psychoeducation, modeling, guided practice, discussion of challenges, and home assignments.
- Home practice and check-ins incorporated to reinforce learning and caregiver well-being
- The CARER intervention session-level structure at the domain level rather than numbered sessions, reflecting flexibility to tailor delivery based on each child’s baseline functioning and caregiver readiness.
- Session content designed to be feasible for primary-care or general medical practitioners, emphasizing clarity, simplicity, and reproducibility.

The CARER program was intentionally designed with sufficient detail and clarity to allow delivery by general medical practitioners or pediatricians, who may be the first or only point of care in resource-limited settings. While we have not formally trained such practitioners in this study, the program’s stepwise structure, explicit examples, and simplified play-based strategies ensure that it can be implemented with minimal specialist expertise. This design choice prioritizes feasibility, replicability, and scalability across low-resource or primary-care contexts.
