# Supplementary material for: CARER program for autism spectrum disorder: a formative qualitative study on developing an early play-based, parent-mediated intervention in the Indian context
Source: Child Adolesc Psychiatry Ment Health. 2026 Jan 16;20:19. doi: 10.1186/s13034-026-01027-2 (PMC12892815; doi:10.1186/s13034-026-01027-2)
Supplement: Supplementary file 4 — Supplementary Material 4. [file 13034_2026_1027_MOESM4_ESM.docx]

**CARER Program for Autism Spectrum Disorder: A Formative Qualitative Study on Developing an Early Play-Based, Parent-Mediated Intervention in the Indian Context.**

**TOYS INVENTORY**

| **Item** | **Primary Purpose/Rationale** | **Approximate Cost Range (INR)** | **Source/ Availability** |
| --- | --- | --- | --- |
| **Functional Toys** | | | |
| Kitchen Set | Pretend play, joint attention, symbolic play | Low – Moderate | Toy stores, online retailers |
| Fruit and Vegetable Toy Models | Pretend play, joint attention, symbolic play | Low | Toy stores |
| Doll House | Symbolic play, joint attention, narrative skills | Low – Moderate | Toy stores, online retailers |
| Doll Figures/Toys | Role play, social reciprocity, emotional modeling | Low | Toy stores |
| Miniature People | Social interaction, pretend play, language modeling | Low | Toy / stationery stores |
| Miniature Animals | Vocabulary development, symbolic play, categorization | Low | Toy stores |
| Vehicles (cars, trucks, boats) | Cause–effect, turn-taking, shared attention | Low | Toy stores |
| Doctor’s Toolkit | Medical play, reducing anxiety, role reversal | Low – Moderate | Toy stores |
| Blocks | Fine motor skills, problem-solving, cooperative play | Low | Toy stores |
| Bowling Set | Gross motor coordination, turn-taking, joint engagement | Low – Moderate | Toy / sports stores |
| Basketball Goal | Gross motor play, regulation, shared play routines | Low – Moderate | Sports / toy stores |
| Toy Phone | Functional communication, imitation, pretend play | Low | Toy stores |
| Toy Camera | Joint attention, imitation, symbolic representation | Low | Toy stores |
| **Expressive/ Constructive Materials** | | | |
| Paper | Expression, choice-making, fine motor skills | Low | Stationery / craft stores |
| Markers and Crayons | Expression, choice-making, fine motor skills | Low | Stationery / craft stores |
| Paints | Sensory regulation, creativity, fine motor skills | Low | Stationery stores |
| Play Doh / Clay | Sensory regulation, creativity, hand strength | Low | Stationery / toy stores |
| Beads | Tactile exploration, regulation | Low | Toy / craft stores |
| Ribbons | Visual tracking, turn-taking, sensory regulation | Low | Craft / stationery stores |
| Pipe Cleaners | Fine motor skills, creativity | Low | Craft stores |
| Confetti | Sensory exploration, shared attention | Low | General / party supply stores |
| Pom Poms | Tactile exploration, regulation | Low | Toy / craft stores |
| Puppets | Emotional expression, communication modeling | Low – Moderate | Craft / toy stores |
| Dress Up Clothes / Hats | Role play, identity exploration, social imitation | Low – Moderate | Toy / costume stores |
| Mr Potato Head | Body awareness, joint attention, symbolic play | Moderate | Toy stores, online retailers |
| Origami Kit | Fine motor skills, sequencing, imitation | Low | Stationery stores, online |
| LEGOs | Problem-solving, shared play, communication | High | Toy stores, online |
| **Sensory Toys** | | | |
| Sand Tray | Sensory exploration, self-regulation | Low | DIY / general stores |
| Water Tray | Sensory exploration, self-regulation | Low | DIY / general stores |
| Moon Sand | Tactile exploration, regulation | Low | Toy / craft stores |
| Snow Mobility | Proprioceptive input, hand strength | Low – Moderate | Surgical suppliers, online |
| Sensory Balls | Tactile input, regulation, focus | Low | Toy / craft stores |
| Theraputty | Proprioceptive input, hand strength | Low – Moderate | Surgical suppliers, online |
| Tangle Toys | Self-regulation, focus | Low | Toy stores, online |
| Sidewalk Chalk | Gross motor play, creativity | Low | Stationery stores |
| Fidget Toys | Tactile input, regulation, focus | Low | Toy / stationery stores |
| Musical Instruments | Auditory engagement, rhythm, joint attention | Low – High | Musical instrument stores / online retailers |
| Slinky | Cause–effect, attention | Low | Toy stores |
| Whirly Wheel | Visual stimulation, motor planning | Low | Toy stores |
| Koosh Ball | Tactile regulation, hand coordination | Low | Toy stores |
| Magnets | Fine motor skills, exploratory play | Low | Toy / stationery / craft stores |
| Punching Bag | Emotional regulation, proprioceptive input | Moderate – High | DIY / sports stores |
| Hula Hoops | Body awareness, coordination | Low | Sports stores |
| Bean Bags | Turn-taking, motor planning | Low – High | Toy / sports stores |
| Balloons | Joint attention, breath control | Low | General / craft stores |
| Bubbles | Shared enjoyment, visual tracking | Low | Toy stores |
| Mini Trampoline | Regulation, balance | Moderate – High | Sports stores |
| **Brain-Based / Cognitive Toys** | | | |
| Brain Puzzles | Problem-solving, persistence, executive functioning | Low | Toy / stationery stores |
| Rubik’s Cube | Planning, visual–spatial skills, frustration tolerance | Low | Toy / stationery Stores |
| **Board / Structured Games** | | | |
| Find It | Visual discrimination, problem solving, attention | Low – Moderate | Toy stores |
| Bop It | Auditory processing, impulse control | Moderate – High | Toy stores |
| Pick-up Sticks | Fine motor control, patience | Low | Toy stores |
| Jacks | Coordination, turn-taking | Low | Toy stores |
| Word Search | Attention, early literacy | Low | Toy / stationery / book stores |
| Word Scramble | Cognitive flexibility, language | Low | Toy / stationery / book stores |
| Barrel of Monkeys | Motor coordination, shared play | Moderate | Toy stores |
| Rush Hour | Planning, problem-solving | Moderate – High | Toy stores |
| Twister | Body awareness, social play | Moderate | Toy stores |
| Spot It | Visual discrimination, attention | Low – Moderate | Toy stores |
| Story Cubes | Narrative skills, imagination, expressive language | Low – Moderate | Toy / stationery stores |
| Memory Games | Working memory, attention, rule-following | Low | Toy stores |
| Chickyboom | Balance, impulse control | Moderate – High | Toy stores |
| Hedbanz | Language, perspective-taking | Moderate | Toy stores |
| Jenga | Turn-taking, impulse control, shared attention | Low – Moderate | Toy stores |

**Table S3: Materials Used in the CARER Program and Their Therapeutic Rationale**

Materials were selected to maximize adaptability, affordability, feasibility, scalability, and sustainability across diverse clinical contexts – consistent with implementation-focused intervention design. They can be used flexibly, with emphasis on caregiver–child interaction styles rather than the toys themselves. Parents were encouraged to adapt activities using household items wherever possible to enhance feasibility and sustainability. Importantly, higher-cost items are largely one-time purchases with repeated usability, whereas most materials used in the program fall within low to moderate cost ranges, enhancing scalability and real-world implementation.

**Intervention Tools and Session-Level Applications**

**Table S4** details the play materials and tools used within the CARER intervention, cross-mapped to session objectives and targeted developmental domains. This table is designed to support implementation and replication by clearly specifying the types of toys and materials employed, their functional role in caregiver–child interactions, and the therapeutic intent guiding their use. Materials were selected based on feasibility, versatility, and availability in typical home and clinical settings, allowing caregivers and practitioners to adapt the intervention across resource contexts while maintaining fidelity to core intervention principles.

| **CARER Program Component** | **Session Focus** | **Materials Used (Examples)** | **Therapeutic Targets** |
| --- | --- | --- | --- |
| **Psychoeducation** | | | |
| Psychoeducation | Understanding autism; caregiver role; developmental differences | Visual charts, handouts, photographs, simple drawings | Knowledge, caregiver empowerment, realistic goal-setting |
| Orientation to Play-Based Intervention | Following the child’s lead; joining play; reinforcement | Blocks, cars, bubbles, balloons, simple puzzles | Joint attention, imitation, positive affect |
| Home-Based Generalization | Embedding strategies into routines | Household items (utensils, containers, clothing) | Skill generalization, caregiver confidence |
| **Socialization** | | | |
| Eye Contact & Joint Attention | Shared gaze; turn-taking; reciprocal interaction | Bubbles, puppets, ball, toy camera, dolls | Joint attention, social reciprocity |
| Imitation & Turn-Taking | Copying actions; waiting; shared rules | Blocks, Jenga, bowling set, basketball goal | Imitation, impulse control, cooperative play |
| Peer Readiness & Peer Play | Playing alongside and with others | Doll house, miniature people, animal figures, board games | Peer interaction, social flexibility |
| **Communication** | | | |
| Speech Stimulation & Modeling | Increasing verbal input; meaningful language | Toy phone, kitchen set, dolls, vehicles | Expressive language, functional communication |
| Requesting & Choice Making | Initiating communication; intentional signaling | Preferred toys kept out of reach, picture cards, toy camera | Initiation, symbolic communication |
| Sequencing & Narratives | Storytelling; recall; sequencing | Story cubes, picture cards, dolls, scrapbook | Narrative skills, temporal understanding |
| **Connection Interventions** | | | |
| Affective Attunement | Physical proximity; shared enjoyment | Balloon games, bubbles, sensory balls | Emotional connection, regulation |
| Body Awareness & Trust | Positive touch; body boundaries | Sand tray, crape paper, magnifying glass, silhouette tracing | Body awareness, relational trust |
| **Restricted Repetitive Behaviors Management** | | | |
| Flexibility & Transitions | Coping with change; reducing rigidity | Visual schedules, blocks, board games | Cognitive flexibility, distress tolerance |
| Redirection & Engagement | Channeling repetitive behaviors into interaction | Vehicles, rhythmic toys, musical instruments | Functional engagement, shared play |
| **Sensory Issues Management** | | | |
| Identification & Modulation | Sensory preferences; graded exposure | Sand tray, water tray, sensory balls, fidget toys | Sensory integration, self-regulation |
| Environmental Adaptation | Reducing overload; supporting focus | Headphones, textured mats, quiet toys | Regulation, attention |

**Table S4: Intervention Tools and Session-Level Applications**
